# Supplementary material for: Room-temperature multiferroicity in sliding van der Waals semiconductors with sub-0.3 V switching
Source: Nat Commun. 2025 Apr 17;16:3648. doi: 10.1038/s41467-025-58009-9 (PMC12006417; doi:10.1038/s41467-025-58009-9)
Supplement: Supplementary file 1 — Supplementary Information [file 41467_2025_58009_MOESM1_ESM.pdf]

Supplementary Information for

Room-temperature multiferroicity in sliding van der Waals semiconductors with sub-0.3 V switching

Rui Chen[1,2,†], Fanhao Meng[1,2,†,*], Hongrui Zhang[1,3,†], Yuzi Liu[4,†], Shancheng Yan[5,†], Xilong Xu[6], Linghan Zhu[6], Jiazhen Chen[1,2], Tao Zhou[4], Jingcheng Zhou[1], Fuyi Yang[1,2], Penghong Ci[1,2], Xiaoxi Huang[1], Xianzhe Chen[1,2], Tiancheng Zhang[1], Yuhang Cai[1,2], Kaichen Dong[1,2], Yin Liu[7], Kenji Watanabe[8], Takashi Taniguchi[8], Chia-Ching Lin[9], Ashish Verma Penumatcha[9], Ian Young[9], Emory Chan[10], Junqiao Wu[1,2], Li Yang[6], Ramamoorthy Ramesh[1,2,11], and Jie Yao[1,2,*]

[1] Department of Materials Science and Engineering, University of California, Berkeley, California 94720, United States.

[2] Materials Sciences Division, Lawrence Berkeley National Lab, Berkeley, California 94720, United States.

[3] Ningbo Institute of Materials Technology & Engineering, Chinese Academy of Sciences, Ningbo 315201, China.

[4] Center for Nanoscale Materials, Nanoscience and Technology Division, Argonne National Laboratory, Lemont, Illinois 60439, United States.

[5] College of Industry-Education Integration, Nanjing University of Posts and Telecommunications, Nanjing 210023, China.

[6] Department of Physics and Institute of Materials Science and Engineering, Washington University in St. Louis, St. Louis, Missouri 63130, United States.

[7] Department of Materials Science and Engineering, North Carolina State University, Raleigh, NC 27606, United States.

[8] National Institute for Material Science, Tsukuba 305-0047, Japan.

[9] Components Research, Intel Corporation, Hillsboro, Oregon 97124, United States.

[10] The Molecular Foundry, Lawrence Berkeley National Laboratory, Berkeley, California 94720, United States.

[11] Department of Physics, University of California, Berkeley, California 94720, United States.

[†] These authors contributed equally.

*Correspondence to: fhmeng@berkeley.edu, yaojie@berkeley.edu.

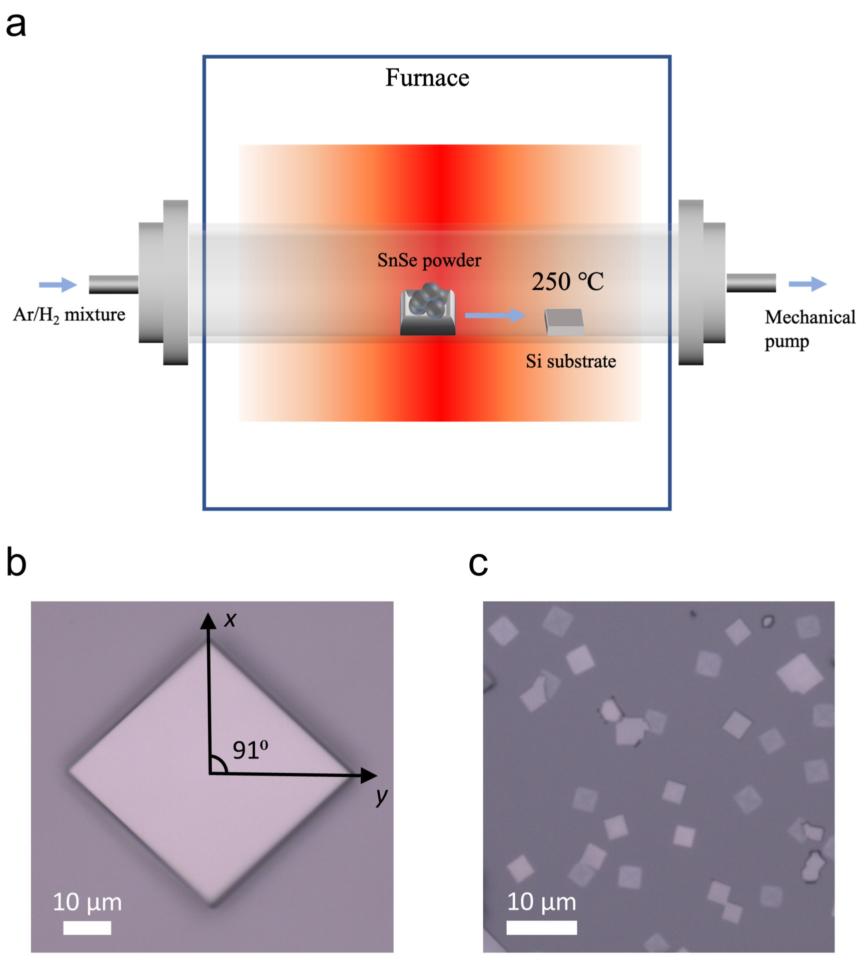

Fig. S1 | PVD synthesis of non-centrosymmetric SnSe crystals. a, Schematic PVD of SnSe crystals directly on Si at low temperature of 250 ºC. This recipe is compatible with CMOS technologies. **b**, A typical thick SnSe single crystal deposited on the Si substrate. Here, *x* and *y* axes correspond to the zigzag and armchair directions of MXs, respectively. **c**, Thin SnSe nanosheets on Si substrate. The thickness of SnSe ranges from 5 to 100 nm.

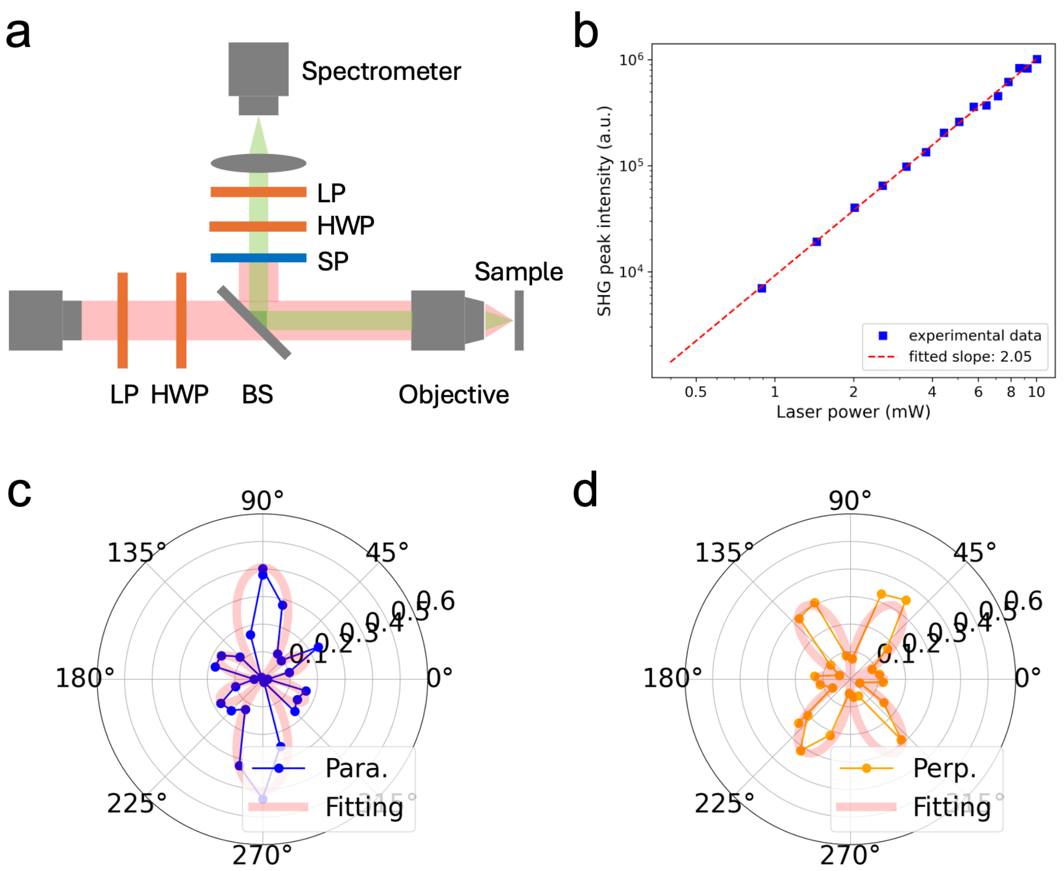

Fig. S2 | Optical SHG of SnSe. a, The schematic set-up to probe the optical SHG. (LP: linear polarizer, HWP: half-wave plate, BS: beam splitter, SP: shortpass filter, L: lens) **b**, Power dependent SHG of a SnSe nanosheet in the log scale. A linear fitting matches perfectly with the experimental data. The slope of 2.05 unambiguously confirms the second order nature of the observed phenomena in Fig. 1e. **c**, **d**, Polarization dependent SHG under 1064 nm CW laser excitation. The two polarizers are set to be parallel (**c**) and perpendicular (**d**) to each other.

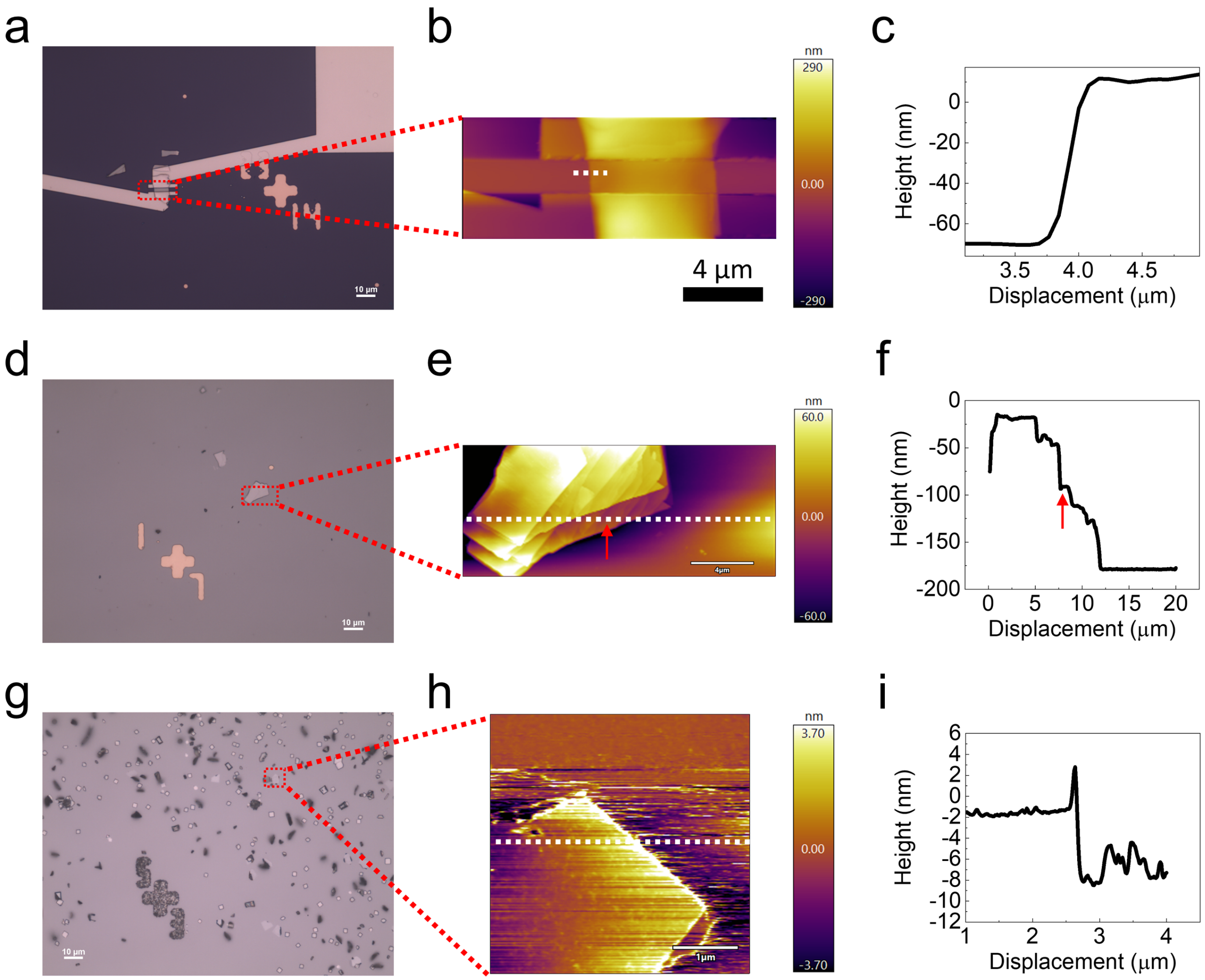

Fig. S3 | Thickness measurements of the PFM samples. a-c, Morphology of the SnSe device tested by IP PFM (Fig. 2b), including the optical microscopy image (**a**), AFM scanning of a local region (**b**), and height profile along the white dashed line in (**b**) (**c**). This IP PFM sample is 81.6-nm-thick. **d-f**, Morphology of the SnSe sample tested by OOP PFM (Fig. 2d). Similarly, the optical image (**d**), AFM mapping (**e**), and height profile (**f**) are incorporated, indicative of the thickness of 87.1 nm. Here, the red arrows mark the local explored region. **g-i**, Optical image (**g**), AFM mapping (**h**), and height profile (**i**) of the ultra-thin SnSe sample probed by OOP PFM (Fig. 2e). The sample thickness is 5 nm.

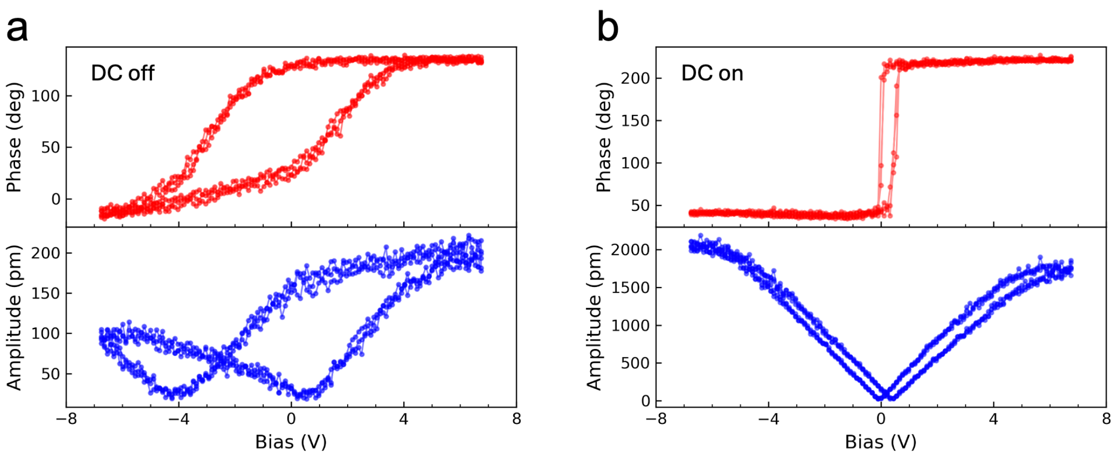

Fig. S4 | Comparison of the PFM curves under "DC off" and "DC on" mode. a, PFM hysteresis measured in "DC off" mode, as shown in Fig. 2d in main text. **b**, PFM curve measured in "DC on" mode. The stark difference verifies the non-volatile nature of the observed ferroelectric switching in Fig. 2.

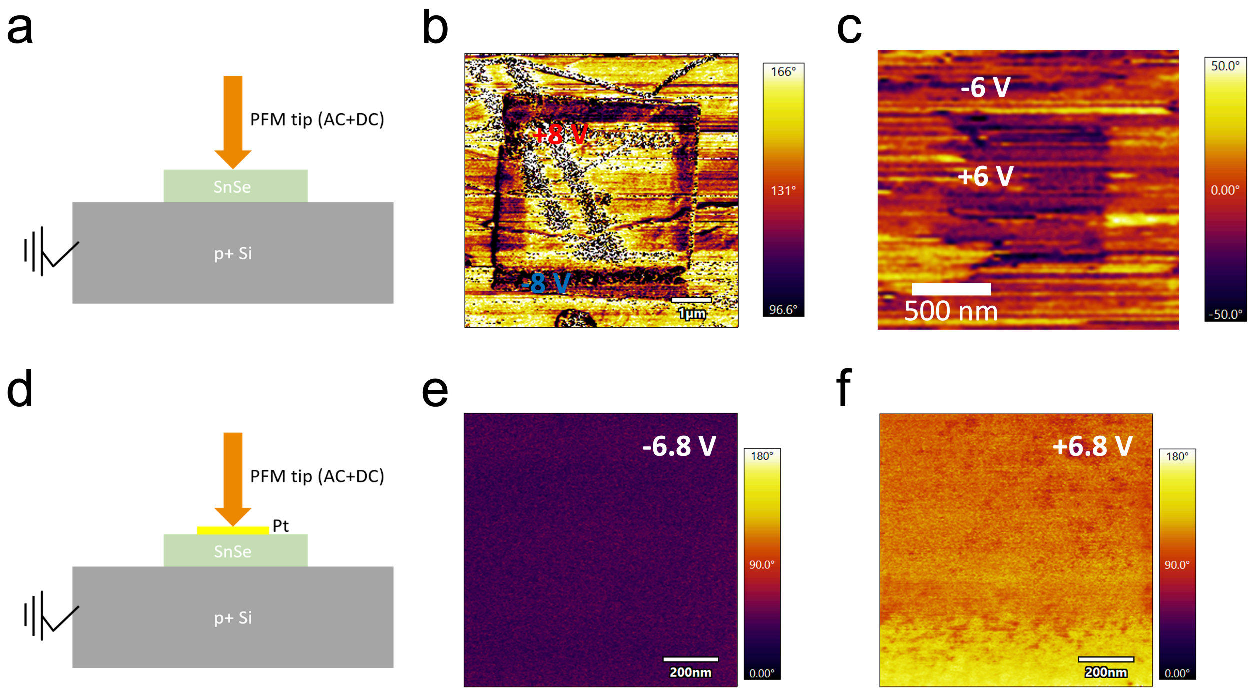

Fig. S5 | DC writing of OOP polarization with PFM tip. a-c, Box-in-a-box patterning of SnSe. The schematic of operation is illustrated in (**a**). One SnSe nanoflake is poled by -8 V first (outer box) and +8 V later (inner box) (**b**) while another sample is poled by -6 V first (outer box) and +6 V later (inner box) (**c**), both of which exhibit strong contrast of phase and great switching capabilities. **d-f**, Area switching through biasing a local point. The schematic diagram is illustrated in (**d**). In order for a better contact between metal and SnSe, a Pt top electrode is patterned on SnSe compared with (**a-c**). The PFM phase mapping of Pt/SnSe after a point switching at -6.8 V (**e**) and +6.8 V (**f**), respectively. The significant contrast between (**e**) and (**f**) suggests the ferroelectric switching throughout the whole sample.

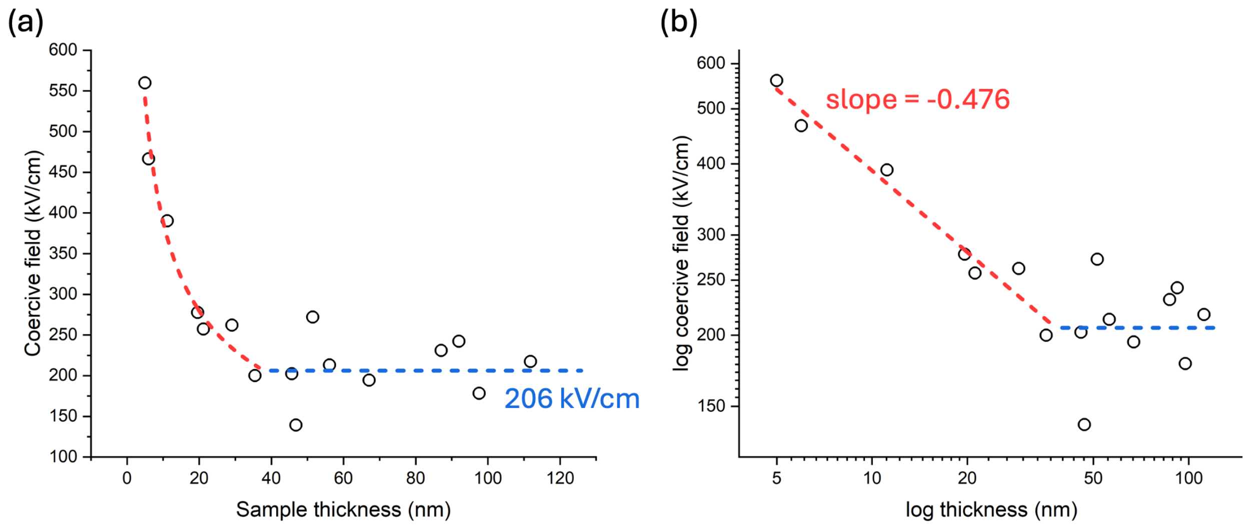

Fig. S6 | Plot of the measured coercive fields with respect to sample thicknesses. a, linear plot. **b**, same plot shown in logarithmic scale. Red dashed line: fitting of the data points below 35 nm thickness, where the coercive field starts to increase with reduced thickness. The slope of -0.476 from log plot indicates a sub-JKD scaling behavior. Blue dashed line: fitting of the data points above 35 nm thickness, where the coercive field is roughly thickness independent.

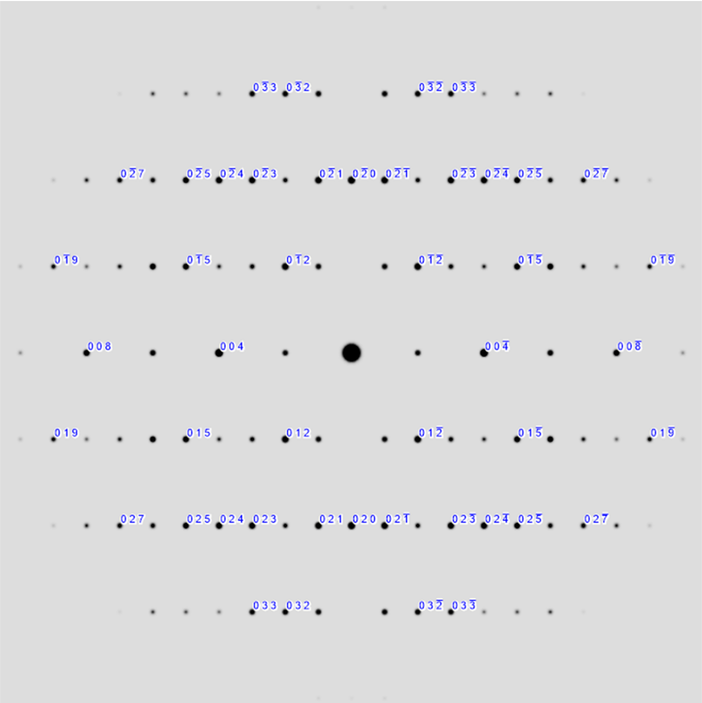

Fig. S7 | Simulated electron diffraction pattern of AB-stacking SnSe along the [010] zone axis. In comparison to both experimental data (Fig. 3b) and simulated (Fig. 3c) AA-SnSe, the simulated AB-SnSe displays more diffraction spots with a higher crystal symmetry. This verifies the AA stacking phase of the synthesized SnSe.

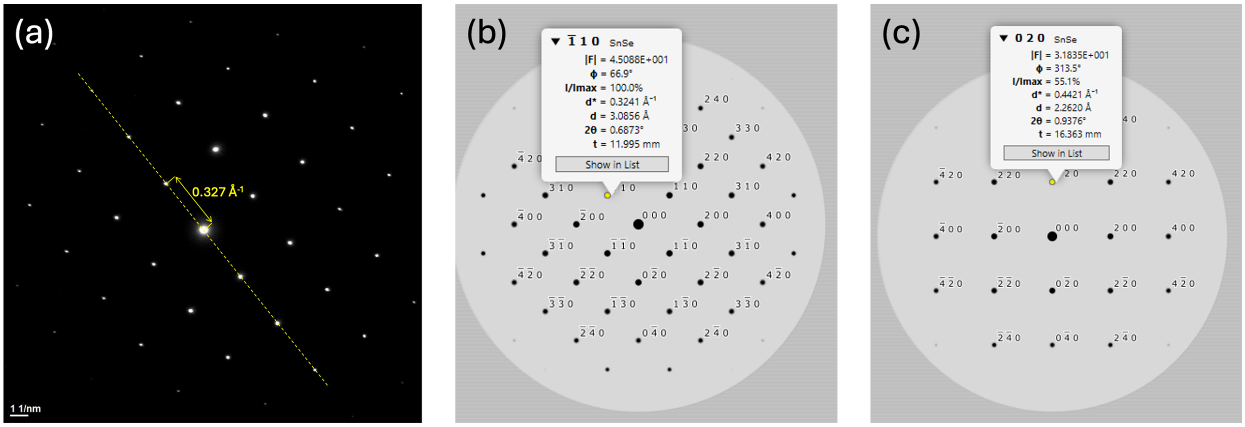

Fig. S8 | Selected area electron diffraction from [001] direction. a, Experimental result. **b**, Simulation of AA stacking, the d spacing matches with experimental results perfectly. **c**, Simulation of AC stacking.

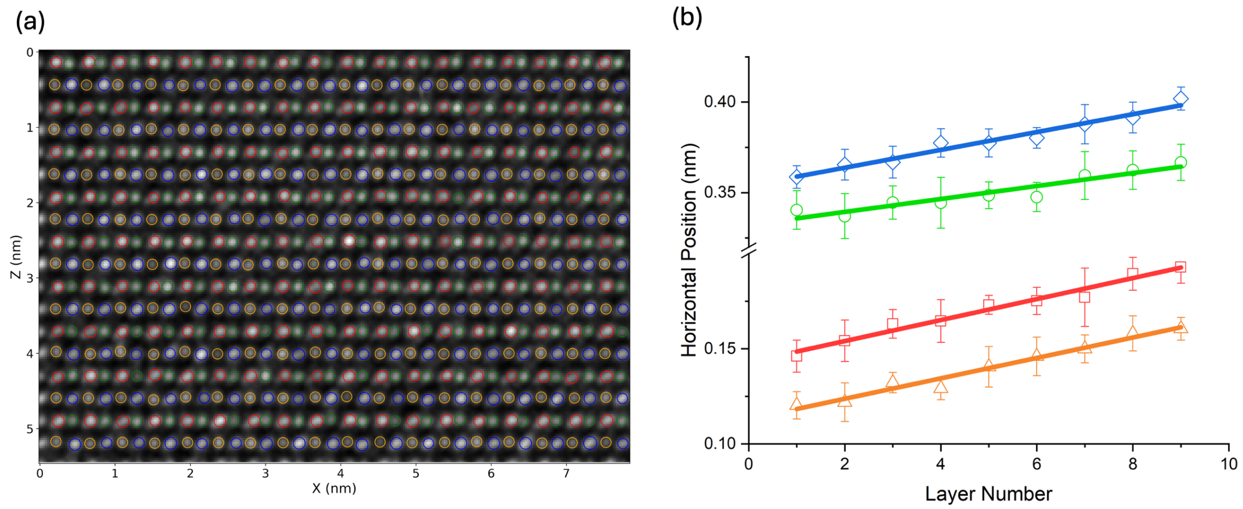

Fig. S9 | TEM analysis of the interlayer sliding in an additional sample image. a, Analysis of the atomic positions from the side view imaged by HRTEM. Atoms are classified and marked by the same color scheme as in Fig. 3d. **b**, The horizontal position of atoms from different layers, confirming a collective interlayer sliding. The error bars indicate the variance of individual atoms' horizontal position within each layer.

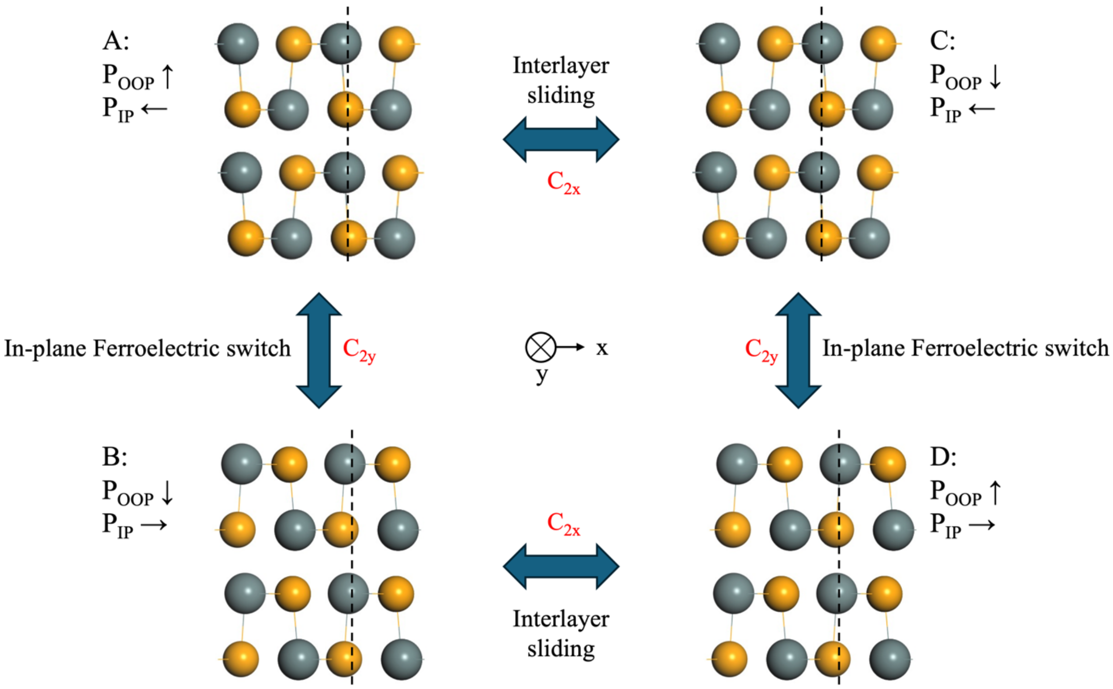

Fig. S10 | Four energy-equivalent polarization states considering different combinations of sliding direction and in-plane polarization direction. The arrows mark the correlation between the states and the corresponding symmetry operations.

Supplementary Note 1: Coupled IP and OOP polarization states related by symmetry operations

The observed OOP polarization is induced by a collective interlayer sliding (the sliding direction is denoted by +*x* or -*x* in Fig. S10). It's worth noting that such sliding is experimentally observed among most of the layers, and the sliding direction is assumed to be fixed in our model, otherwise the energy barrier of reversing the overall sliding direction would be too high. In this case, the sign of the OOP polarization (+*z* or -*z*) is determined by both the IP polarization state and the sliding direction. For example, if the sliding direction is -*x* (state A and B in Fig. S10), then the IP polarization $-P_x$ is locked with OOP polarization $+P_z$ (state A), and vice versa (state B). From the symmetry point of view, such IP and OOP coupling are described by a C_{2y} operation, which always flips both IP and OOP polarizations simultaneously. The role of sliding direction can be described by a C_{2x} operation, which preserves the IP polarization but flips the OOP one.

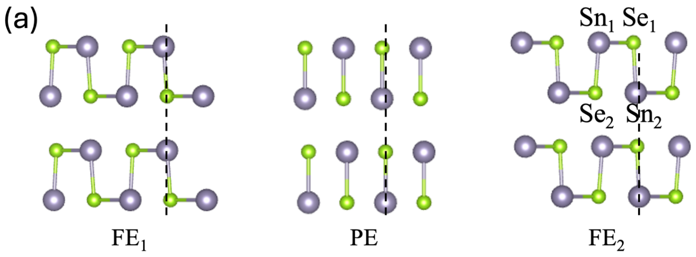

(b)

Atom	Shift (Å)
Sn$_1$	0.064
Sn$_2$	0.2
Se$_1$	0.15
Se$_2$	0.13
Average/layer	0.136

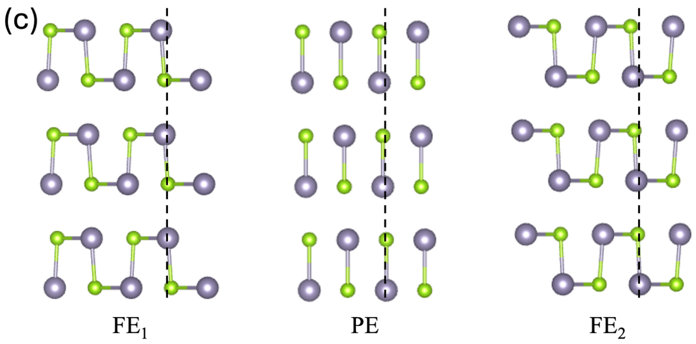

(d)

Atom	Shift (Å)
Sn$_1$	0.1955
Sn$_2$	0.02284
Se$_1$	0.09226
Se$_2$	0.13046
Average/layer	0.055

Fig. S11 | Calculated atomic shift for the 4 types of atoms within a unit cell. After applying the interlayer sliding in the ab-initio calculation, a structural relaxation confirms that the atomic shifts for different type of atoms are different. This result agrees well with the HRTEM observation. **a-b**, Bilayer model. **c-d**, Tri-layer model.

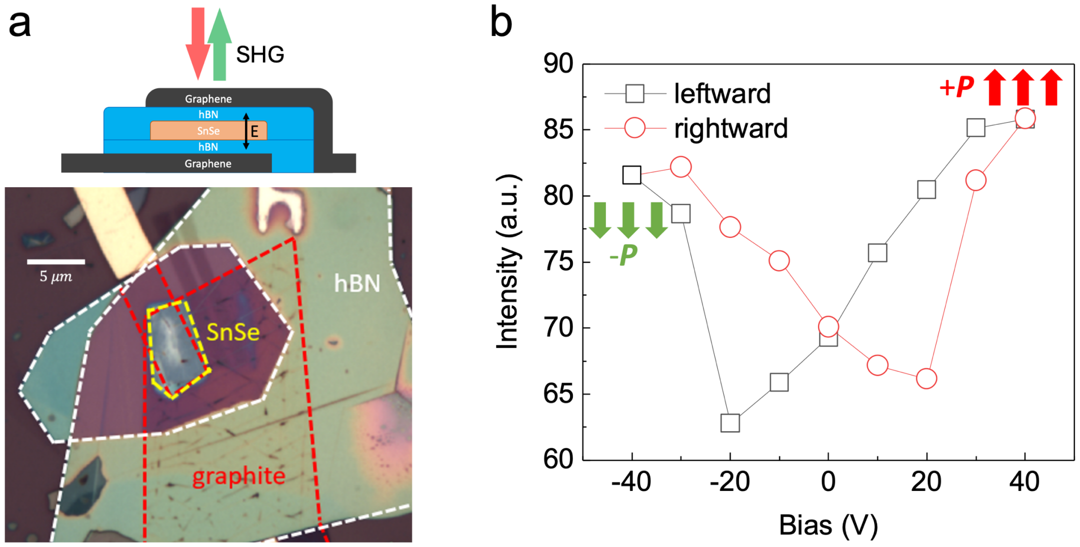

Fig. S12 | Tuning the SHG of SnSe with a vertical electric field. a, Optical microscopy image of the device. The top panel shows a schematic of the graphite/hBN/SnSe/hBN/graphite heterostructure, generating a vertical E-field. In-situ SHG signals can be probed as the bias voltage is swept. **b**, IP SHG intensity as a function of OOP bias voltage. Here, the arrows schematically describe the ferroelectric polarization orientations in SnSe. This hysteretic modulation of SHG intensity offers solid evidence of the intrinsic coupling between IP and OOP ferroelectric polarizations.

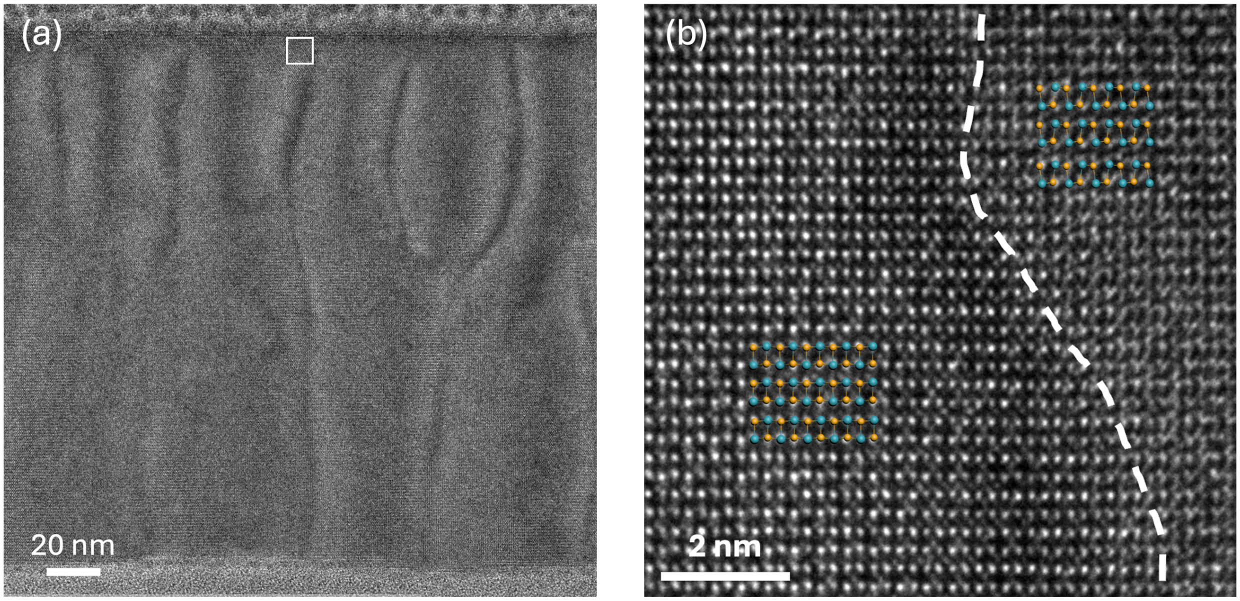

Fig. S13 | Ferroelastic domains imaged by TEM. a, Low-magnification side view showing stripes-like contrast. The white box marks the region where the HRTEM image in panel b is taken. **b**, HRTEM image of the selected region under higher magnification. The white dashed line indicates the domain boundary.

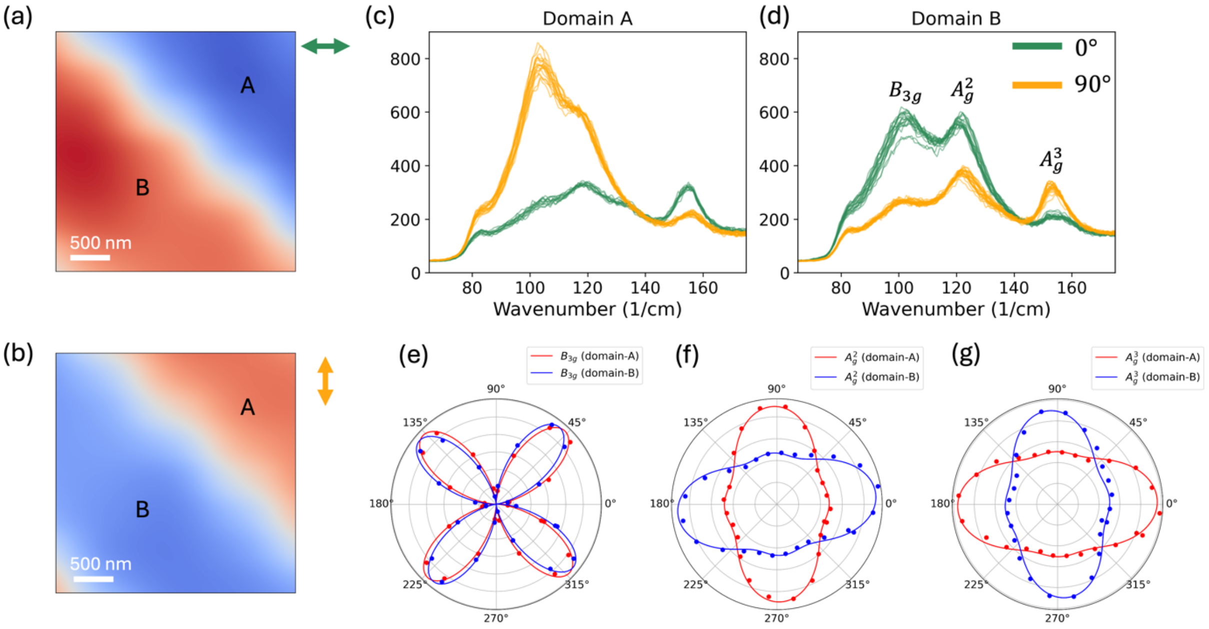

Fig. S14 | Linearly polarized Raman mapping around a domain boundary with 532 nm excitation. a-b, Intensity map of A_g^2 peak (~120 cm^{-1}) with the polarizers set to horizontal (a) and vertical (b) position. Upon rotating the polarization from 0° to 90°, the intensity contrast between domain A and domain B flips. **c-d**, Full Raman spectra in domain A and B under 0° and 90° polarization angles, clearly exhibiting the discrepancy due to crystalline anisotropy. **e-g**, Polarization-angle-dependent polar plots of the Raman B_{3g}, A_g^2 and A_g^3 peaks, taken from a spot in domain A (red curve) and another spot in domain B (blue curve). It is evident that the lattice orientations of the two domains are ~87° apart, one corresponding to the armchair-like and the other zigzag-like.

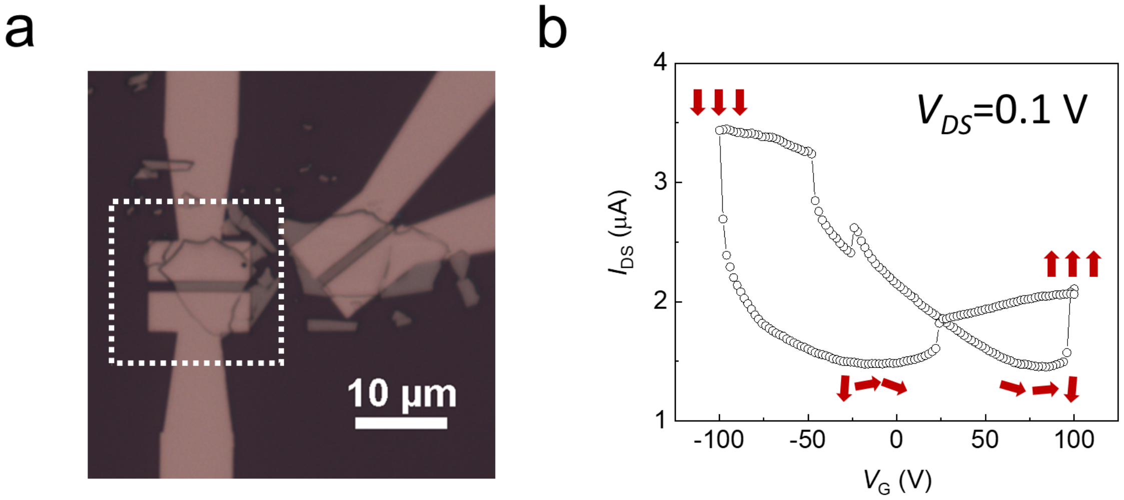

Fig. S15 | Transfer curves of ferroelectric SnSe. a, Optical microscopy image of a two-terminal transistor based on the AA-SnSe/300 nm SiO_2/Si (white dashed frame). **b**, Nonvolatile I_{DS}-V_G curves of the SnSe device in (**a**) at the V_{DS} of 0.1 V. Red arrows schematically describe the ferroelectric polarization near the interface of SnSe and SiO_2. This unconventional butterfly behavior can be attributed to the fact that V_G flips the interfacial ferroelectric polarization and thus manipulates the local charge concentration, which mediates the horizontal electric conductivity in a hysteretic manner.
