# Supplementary material for: Room-temperature multiferroicity in sliding van der Waals semiconductors with sub-0.3 V switching
Source: Nat Commun. 2025 Apr 17;16:3648. doi: 10.1038/s41467-025-58009-9 (PMC12006417; doi:10.1038/s41467-025-58009-9)
Supplement: Supplementary file 2 — Reporting Summary [file 41467_2025_58009_MOESM2_ESM.pdf]

natureresearch

Corresponding author(s): Fanhao Meng, Jie Yao

Lasing Reporting Summary

Nature Research wishes to improve the reproducibility of the work that we publish. This form is intended for publication with all accepted papers reporting claims of lasing and provides structure for consistency and transparency in reporting. Some list items might not apply to an individual manuscript, but all fields must be completed for clarity.

For further information on Nature Research policies, including our data availability policy, see Authors & Referees.

Experimental design

Please check: are the following details reported in the manuscript?

1. Threshold

Plots of device output power versus pump power over a wide range of values indicating a clear threshold

☒ Yes ☐ No

Although this work doesn't involve a lasing device, we do provide the SHG power versus pump power in Supplementary Fig. S2b

2. Linewidth narrowing

Plots of spectral power density for the emission at pump powers below, around, and above the lasing threshold, indicating a clear linewidth narrowing at threshold

☐ Yes ☒ No

This work doesn't involve a lasing device, but uses commercial laser systems to perform SHG and Raman measurements. The linewidth narrowing is not relevant to those experiments.

Resolution of the spectrometer used to make spectral measurements

☐ Yes ☒ No

We didn't perform linewidth measurement as explained above, so the resolution of the spectrometer is not relevant.

3. Coherent emission

Measurements of the coherence and/or polarization of the emission

☒ Yes ☐ No

Although this work doesn't involve a lasing device, we do provide polarization-resolved measurement of the SHG and Raman emission as shown in Fig. 1e, Fig. S2c-d, and Fig. S14.

4. Beam spatial profile

Image and/or measurement of the spatial shape and profile of the emission, showing a well-defined beam above threshold

☐ Yes ☒ No

This work doesn't involve a lasing device, but uses commercial laser systems to perform SHG and Raman measurements. The beam spatial profile is not relevant to those experiments.

5. Operating conditions

Description of the laser and pumping conditions
Continuous-wave, pulsed, temperature of operation

☒ Yes ☐ No

The operating conditions are provided in Methods section, under "SHG measurements and calculations".

Threshold values provided as density values (e.g. W cm^{-2} or J cm^{-2}) taking into account the area of the device

☐ Yes ☒ No

This work doesn't involve a lasing device, so the threshold values are not relevant.

6. Alternative explanations

Reasoning as to why alternative explanations have been ruled out as responsible for the emission characteristics
e.g. amplified spontaneous, directional scattering; modification of fluorescence spectrum by the cavity

☐ Yes ☒ No

This work doesn't involve a lasing device, so alternative explanations are not evaluated.

7. Theoretical analysis

Theoretical analysis that ensures that the experimental values measured are realistic and reasonable
e.g. laser threshold, linewidth, cavity gain-loss, efficiency

☐ Yes ☒ No

This work doesn't involve a lasing device, so we didn't analyze such information. The theoretical analysis for SHG and inversion symmetry breaking is presented in main text.

8. Statistics

Number of devices fabricated and tested

☐ Yes ☒ No

This work doesn't involve any lasing devices. Only commercial laser systems are used.

Statistical analysis of the device performance and lifetime (time to failure)

☐ Yes ☒ No

This work doesn't involve any lasing devices. Only commercial laser systems are used.
